# Cutaneous Regeneration Mechanism of β-Sheet Silk Fibroin in a Rat Burn Wound Healing Model

**DOI:** 10.3390/polym13203537

**Published:** 2021-10-14

**Authors:** Kai-Chieh Chou, Chun-Ting Chen, Juin-Hong Cherng, Ming-Chia Li, Chia-Cheng Wen, Sheng-I Hu, Yi-Wen Wang

**Affiliations:** 1 Graduate Institute of Life Sciences, National Defense Medical Center, Taipei 114, Taiwan; jeffrey8464@gapps.ndmctsgh.edu.tw (K.-C.C.); i72bbb@gmail.com (J.-H.C.); 2 Division of Gastroenterology and Hepatology, Department of Internal Medicine, Tri-Service General Hospital Penghu Branch, National Defense Medical Center, Taipei 114, Taiwan; gp0921543gp@yahoo.com.tw; 3 Division of Gastroenterology and Hepatology, Department of Internal Medicine, Tri-Service General Hospital, National Defense Medical Center, Taipei 114, Taiwan; 4 Laboratory of Adult Stem Cell and Tissue Regeneration, National Defense Medical Center, Taipei 114, Taiwan; 5 Department and Graduate Institute of Biology and Anatomy, National Defense Medical Center, Taipei 114, Taiwan; 6 Department of Biological Science and Technology, Center For Intelligent Drug Systems and Smart Bio-Devices (IDS2B), National Yang Ming Chiao Tung University, Hsinchu 300, Taiwan; mingchiali@g2.nctu.edu.tw; 7 Division of Colon and Rectal Surgery, Department of Surgery, Tri-Service General Hospital, National Defense Medical Center, Taipei 114, Taiwan; wjason@mail2000.com.tw (C.-C.W.); roger720922@msn.com (S.-I.H.)

**Keywords:** burn injury, wound healing, β-sheet silk fibroin, cell migration, integrin, extracellular matrix protein, *Bombyx mori*

## Abstract

Therapeutic dressings to enhance burn wound repair and regeneration are required. Silk fibroin (SF), a natural protein, induces cell migration and serves as a biomaterial in various dressings. SF dressings usually contain α-helices and β-sheets. The former has been confirmed to improve cell proliferation and migration, but the wound healing effect and related mechanisms of β-sheet SF remain unclear. We investigated the effects of β-sheet SF in vivo and in vitro. Alcohol-treated α-helix SF transformed into the β-sheet form, which promoted granulation formation and re-epithelialization when applied as lyophilized SF dressing (LSFD) in a rat burn model. Our in vitro results showed that β-sheet SF increased human dermal fibroblast (HDF) migration and promoted the expression of extracellular matrix (ECM) proteins (fibronectin and type III collagen), matrix metalloproteinase-12, and the cell adhesion molecule, integrin β1, in rat granulation tissue and HDFs. This confirms the role of crosstalk between integrin β1 and ECM proteins in cell migration. In summary, we demonstrated that β-sheet SF facilitates tissue regeneration by modulating cell adhesion molecules in dermal fibroblasts. LSFD could find clinical application for burn wound regeneration. Moreover, β-sheet SF could be combined with anti-inflammatory materials, growth factors, or antibiotics to develop novel dressings.

## 1. Introductions

Burn injuries cause severe damage to the epidermis, dermis, and underlying tissue, often resulting in physical and mental illnesses [1]. Ideally, burn wound dressings should be infection-resistant, retain moisture, and facilitate tissue regeneration [2]. Biomaterials applied in wound dressings have been reported to induce cell migration, re-epithelialization, and granulation tissue formation during the proliferation phase of cutaneous wound healing [3,4,5].

Silk, derived from *Bombyx mori* cocoons, is a well-known and favored natural proteinaceous fiber with a long history of utilization in textiles. One of its main components is silk fibroin (SF). The structure of SF is characterized by macromolecules with three amino acids in repeating sequences (Gly–Ser–Gly–Ala–Gly–Ala) [6,7]. SF is biodegradable and biocompatible, and has been applied in surgical sutures, skincare products [8,9], and tissue engineering in the skin [10], cartilage [11], and corneal repair [12]. Additionally, SF has been developed into several forms of dressings, including sponges, films, scaffolds, electrospun nanofibers, and hydrogels [13,14]. Silk fibroin exists in alpha-helix and beta-sheet form, both of which are highly biocompatible and can also promote tissue regeneration [13,15]. However, it is difficult for amorphous silk fibroin (α-helix or random coil) to form stable scaffolds [16,17]. β-sheet silk fibroin provides good mechanical properties and structure for cell attachment; moreover, it shows a nontoxic influence on fibroblasts and stem cells [18,19], facilitation in collagen deposition during bone regeneration [19], and promotion for angiogenesis during wound healing [20]. For building a structurally stable scaffold and a biomaterial for wound dressing, the β-sheet form SF was prioritized [21,22]. It has been reported that lyophilized SF scaffolds possess biocompatible properties and pores optimized for cell growth [19,23]. Alcohol treatment of lyophilized SF induces the transition of the α-helices to β-sheets to form a stable wound dressing [24]. We aimed to evaluate whether this lyophilized SF dressing (LSFD) could promote burn wound healing.

During the proliferation phase, extracellular matrix (ECM) deposition is essential for cell adhesion, which promotes cell migration during wound re-epithelialization and granulation. Natural SF has a hydrophilic soluble α-helix form and a self-crystalline insoluble β-sheet form [6,25]. It has been reported that α-helix SF induces the gene expression of the mechanistic target of rapamycin (mTOR) axis and cell adhesion molecules, and elevates ECM protein expression via the nuclear factor kappa B (NF-κB) signaling pathway in human dermal fibroblasts (HDFs) [26]. However, LSFD is mainly composed of β-sheet SF, and the effect and related mechanism of β-sheet SF on ECM expression and cell migration during wound healing remain unclear.

Integrins, which are cell adhesion molecules, are cell surface receptors and are composed of α- and β-subunits that specifically contact ECM proteins [27]. This facilitates multiple cellular activities, including cell migration and cytoskeleton modulation [27,28]. In vitro studies have shown that SF increases integrin expression in human umbilical vein endothelial cells [26,29,30]. These in vitro data showed that SF treatment potentially promotes cell motility by increasing integrin expression and ultimately results in wound regeneration. However, this inference has not been confirmed in skin cells and animal wound model. Therefore, we determined integrin expression after β-sheet SF treatment in HDFs and burn wounds in rats.

In this study, we aimed to investigate the effect and underlying mechanism of β-sheet SF in HDFs and keratinocytes, and evaluate the effect of lyophilized SF dressing (LSFD) in a rat burn wound healing model by analyzing granulation tissue formation and wound re-epithelialization. These results suggest that LSFD could be applied clinically to improve burn wound regeneration. Moreover, the β-sheet SF structure could lend stability to combinations with other anti-inflammatory materials, growth factors, or antibiotic reagents, to develop novel dressings with ideal characteristics to promote burn wound regeneration.

## 2. Materials and Methods

### 2.1. SF Solution Extraction and Characterization

The extraction of the SF aqueous solution complied with previous research [26,31,32]. Silk cocoons of *B. mori* were purchased from Danee Company (New Taipei City, Taiwan). Briefly, to remove the silk sericin coating, the cocoons were cut into pieces and degummed for 30 min with a 0.02 M Na_2_CO_3_ solution at 100 °C. After washing with distilled water and drying overnight in an oven at 37 °C, degummed fibers were dissolved in a 9.3 M LiBr solution at 60 °C for 6 h. Until the degummed fibers were solubilized, this silk solution was dialyzed in distilled water at room temperature for three days using dialysis membranes (Cellu-Sep; nominal molecular weight cutoff: 12,000–14,000; Interchim, Montluçon, France). The dialyzed silk solution was centrifuged at 500 rpm for 20 min. The final concentration of SF solution was approximately 5–6% (*w*/*v*), determined by weighing the residual solid after oven-drying at 37 °C. The SF aqueous solution was stored at 4 °C until required for further use. The SF aqueous solution was coated onto a quartz plate. After evaporation, a 75% ethanol solution was added. Circular dichroism (CD) spectra were measured and recorded using a J-1700 CD spectrophotometer (Jasco Inc., Easton, MD, USA).

### 2.2. LSFD Preparation

The preparation of LSFD by lyophilization was performed using a method we had published before [19]. After freeze-drying based on temperature modulation, LSFD was obtained via β-sheet crystallization using a 75% ethanol solution treatment. The ethanol solution was removed by complete evaporation in a hood. Then, the LSFDs were stored at room temperature until required. The pore size is approximately 170 µm in diameter, and 75% of pores are interconnected [19].

### 2.3. Rat Burn Model

Seven Sprague–Dawley rats (150–200 g; 7 weeks old) were purchased from Bio-LASCO Co., Ltd. (Taipei City, Taiwan). The burning procedure was performed according to Wang et al. [33], and all animal experiments were approved by and conducted in accordance with the National Defense Medical Center Institutional Animal Care and Use Committee (IACUC-20-016). A total of 12 female rats were used in this study: five rats for the condition test and seven for the repeat experiment and data analysis. Before the burn wound was created using a 2-cm diameter brass block at 100 °C for 10 s, the rats were shaved dorsally and received anesthesia via intramuscular injection of Zoletil 50 (20 mg/kg; Virbac, Carros, France) in the calf area. Two parallel burn wounds were created on the back of each rat; the distance between the two wound edges was approximately 3 cm. The heated brass block caused second-degree burn wounds [34]; the wounds were covered with gauze and Tegaderm transparent film dressing (3M Health Care, St. Paul, MN, USA) to prevent scratching. All the rats were housed individually in cages under aseptic conditions after surgery.

After wounding on day 0, debridement was performed the next day (day 1). The two burn wounds on each rat were covered with gauze (control group) or LSFD (LSFD group), respectively. The animals were kept warm with an electrically heated blanket during the surgery, and received daily intramuscular injections of ketoprofen (2.5 mg/kg; Astar, Hsinchu, Taiwan) for analgesia and cefazolin (25 mg/kg; China Chemical & Pharmaceutical, Tapei City, Taiwan) to prevent infection after surgery. Each wound was disinfected daily with iodine solution and cleaned with sterile normal saline, cotton swabs, and gauze.

### 2.4. Wound Closure Analysis

The burn wounds were recorded with a digital camera on days 0, 1, 3, 7, and 10, and the wound area was measured using ImageJ software (19 Janruary 2021 accessed, https://imagej.nih.gov/ij/; NIH, Bethesda, MD, USA). The gauze and LSFD were replaced after the wound was recorded. The wound closure rate was computed by two blinded evaluators and expressed as the percentage of the initial wound size.

### 2.5. Histological Analysis

All rats were euthanized on day 10. The wound area and surrounding normal tissue were harvested and cut into halves; one piece was immediately fixed in 4% paraformaldehyde solution for section staining, and the other was frozen in liquid nitrogen for Western blot analysis. After fixation, the tissues were embedded in paraffin. The sections (5 μm) were stained with hematoxylin and eosin (H&E) (Merck Millipore, Darmstadt, Germany) and Masson’s trichrome stain kit (ScyTek Laboratories, Logan, UT, USA), following the manufacturer’s protocol. The stained sections were viewed and photographed using an Axio Scan Z1 slide scanner (ZEISS, Oberkochen, Germany).

### 2.6. Immunohistochemistry (IHC) Staining

The 5 μm tissue sections were dewaxed with xylene (Ferak Berlin, Germany) and rehydrated. Each section was blocked with 10% normal goat serum in phosphate-buffered saline (PBS) for 1 h and then incubated with the primary antibody in blocking solution at room temperature for 1 h. The following primary antibodies were used for staining: mouse anti-vitronectin, mouse anti-fibronectin (FN), mouse anti-integrin α1, mouse anti-integrin α2, mouse anti-integrin α5, mouse anti-integrin αv, mouse anti-integrin β1, mouse anti-integrin β3, (sc-74484, sc-8422, sc-271034, sc-74466, sc-376199, sc-376156, sc-374429, and sc-365679, respectively, 1:1000, Santa Cruz Biotechnology, Inc., Dallas, TX, USA), mouse anti-collagen I, rabbit anti-collagen III, rabbit anti-matrix metalloproteinase-1 (MMP-1), rabbit anti-MMP-12, rabbit anti-MMP-13, mouse anti-α-smooth muscle actin (SMA) (GTX26308, GTX102997, GTX00674, GTX100704, GTX100665, GTX629702, respectively; 1:1000; GeneTex, Irvine, CA, USA). After removing the immersing solution, all sections were washed three times for 5 min with PBS and then incubated with the second antibody containing biotin. After washing, sections were soaked using an ABC kit (Vector Laboratories, Inc., Burlingame, CA, USA) for 1 h and visualized using 3,3′-diaminobenzidine solution containing 3% hydrogen peroxide. All the tissue sections were then counterstained with hematoxylin and photographed using an Axio Scan Z1 slide scanner (ZEISS).

### 2.7. Immunofluorescence Staining

The frozen tissue sections (20 μm) were washed with PBS and blocked with 0.1% goat serum in PBS for 30 min. The keratin-14 (K14) antibody diluted in blocking solution was used at a 1:1000 dilution for 1 h. After washing with PBS, the tissue samples were incubated with goat anti-mouse IgG secondary antibody (Alexa Fluor 488, 1:1000; Invitrogen, Waltham, MA, USA) for 1 h. Finally, the samples were mounted in Permount Mounting Medium (SP15-500, Fisher Scientific, Waltham, MA, USA) and photographed using an Axio Scan Z1 slide scanner (ZEISS).

### 2.8. Fibroblast Isolation and Cell Culture

Primary cultured HDFs were obtained by circumcision surgery. Foreskin collection was approved by the Tri-Service General Hospital Institutional Review Board. Tissue samples were prepared as described by Rnjak et al. [35]. The separated cells were maintained in Dulbecco’s modified Eagle medium (DMEM; Gibco, Waltham, MA, USA) supplemented with 10% fetal bovine serum (FBS) and 1% penicillin-streptomycin. All cell experiments were performed using primary cultured HDFs at passages 2–5.

### 2.9. HaCaT Cell Culture

The HaCaT immortal human keratinocyte cell line was purchased from CLS Cell Lines Service GmbH (#300493; Eppelheim Germany). HaCaT cells were cultured in DMEM (3.7 g/L NaHCO_3_ and 110 mg/L sodium pyruvate) (Gibco) supplemented with 10% FBS, 1% penicillin–streptomycin, and 2 mM L-glutamine in a CO_2_ incubator at 37 °C.

### 2.10. Cell Migration

A culture insert (#80209; ibidi GmbH, Gräfelfing, Germany) was placed in each well of a 24-well culture plate. For the SF group, the inside bottom area of the culture insert was coated in aqueous solution of SF (0.1 μg/cm^2^). After evaporation, a 75% ethanol solution was added for to induce β-sheet formation [24]. The control group culture insert was not treated with SF coating. Primary HDFs and HaCaT cells were maintained in the culture medium with 1% FBS for 24 h and were seeded outside the culture-inserts at a density of 1 × 10^6^ cells per well. Once all the cells were attached to the plate, the cell-migrating area was observed and recorded at 24, 72, and 168 h, after removing the culture-inserts.

### 2.11. Western Blot

Primary cultures of HDFs (2 × 10^5^ cells per well) were seeded in a six-well culture plate coated with or without SF. After treatment for 168 h, the cultured cells were harvested directly using a radioimmunoprecipitation assay lysis buffer system (sc-24948; Santa Cruz Biotechnology). The rat wound tissue was homogenized in lysis buffer. Cell and tissue protein concentrations were determined using a Coomassie protein assay reagent (Thermo Fisher Scientific, Waltham, MA, USA). For sodium dodecyl sulfate-polyacrylamide gel electrophoresis, the protein samples (15 μg) were loaded on 10% SDS–PAGE gel and transferred to an Immobilon-E polyvinylidene fluoride membrane (Merck Millipore, Burlington, MA, USA) by electroblotting. The membranes were incubated with the primary antibodies mentioned above (Section 2.6). Other primary antibodies included rabbit anti-p-mTOR, rabbit anti-mTOR, rabbit anti-p-NF-κB, rabbit anti-NF-κB, mouse anti-nuclear factor of kappa light polypeptide gene enhancer in B-cells inhibitor, alpha (IκBα), rabbit anti-phosphorylated-extracellular signal-regulated kinase 1/2 (ERK1/2), rabbit anti-ERK1/2, rabbit anti-phosphorylated-c-Jun N-terminal kinase (JNK), rabbit anti-JNK, rabbit anti-phosphorylated-c-Jun, rabbit anti-c-Jun (#5536, #2972, #3033, #8242, #4814, #4370, #46955, #4668, #9252, #9261, #9165, respectively; 1:1000, Cell Signaling Technology, Inc., Danvers, MA, USA), mouse anti-phosphorylated-IκBα, mouse anti-glyceraldehyde 3-phosphate dehydrogenase (GAPDH), mouse anti-α-actinin (sc-8404, sc-32233, sc-17829, respectively; 1:1000; Santa Cruz Biotechnology). The following secondary antibodies were used: goat anti-rabbit IgG antibody, mouse IgG antibody (GTX213110-01 and GTX213111-01, respectively; 1:1000; GeneTex). Protein signals from the membranes were detected with Amersham ECL reagent (RPN2106; Cytiva, Marlborough, MA, USA) using a chemiluminescent imaging and analysis system (MiniChemi 610; Sage Creation Science, Beijing, China). ImageJ software was used to detect the protein signal values.

### 2.12. Statistical Analysis

Statistical analyses were performed using GraphPad Prism 8 software (GraphPad Software, Inc., San Diego, CA, USA). All data are expressed as the mean ± standard error of the mean. Significant differences between groups were analyzed using the one-tailed paired *t*-test. Statistical significance was set at *p* < 0.05.

## 3. Results

### 3.1. Preparation and Analysis of Ethanol-Treated LSFD

**Figure 1:**
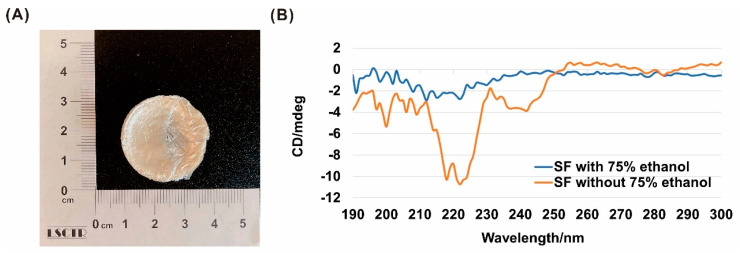
(**A**) Lyophilized silk fibroin dressing (LSFD) for the in vivo study; (**B**) circular dichroism (CD) spectra of SF treated with or without 75% ethanol.

We used LSFD treated with 75% ethanol for the animal study (Figure 1A). The micro characterization including scanning electron microscopy image of LSFD was showed in our previously published paper [19]. For the CD spectra of SF structure determination, we measured SF with or without 75% ethanol treatment. The secondary structure of the untreated SF coating is represented by two peaks between 208–230 nm and the sloping ellipticity between 230–240 nm, while SF treated with 75% ethanol showed a simple curve between 208–230 nm (Figure 1B). Our investigation showed that SF treated with 75% ethanol had more β-sheets than untreated SF coatings, in accordance with previous research [36,37,38,39].

### 3.2. β-Sheet LSFD Promoted In Vivo Tissue Regeneration

**Figure 2:**
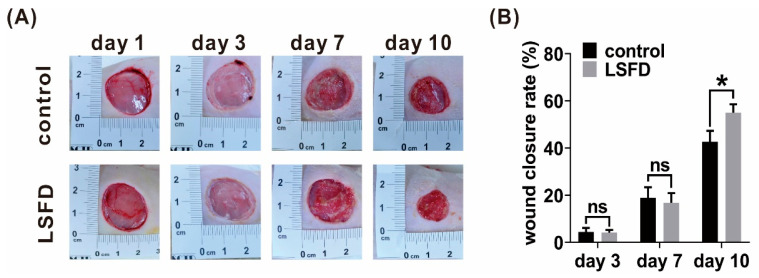
In vivo wound repair evaluation of β-sheet lyophilized silk fibroin dressing (LSFD)-treated burn injuries in rats. (**A**) The photographic appearance of burn wounds in β-sheet LSFD and control groups on day 1, 3, 7, and 10 post-wounding. All animals received wound debridement on day 1. (**B**) Quantitative representation of the wound closure rate. Data are presented as the mean ± standard error of the mean (*n* = 7; * *p* < 0.05, ns: not significant).

The in vivo wound closure rate was evaluated (Figure 2) to investigate whether LSFD promoted wound healing. Two burn wounds were created on each rat’s dorsal skin and debrided the next day. One of the burn wounds on each rat was treated with β-sheet LSFDs after debridement, while the other wound, treated with medical gauze, served as the control. Notably, the LSFD group had minor wounds on day 10 compared with the control group (Figure 2A). Furthermore, the wound healing rate in the LSFD group was increased by 12% vs. the control group (*p* < 0.05) (Figure 2B). However, there was no significant difference in wound contraction between the LSFD and control groups on days 3 and 7 (Figure 2). In summary, LSFD promoted burn wound repair in the late phase of wound healing, known as the proliferation phase.

### 3.3. β-Sheet LSFD Accelerated Re-Epithelialization and Granulation Formation in Burn Wounds

**Figure 3:**
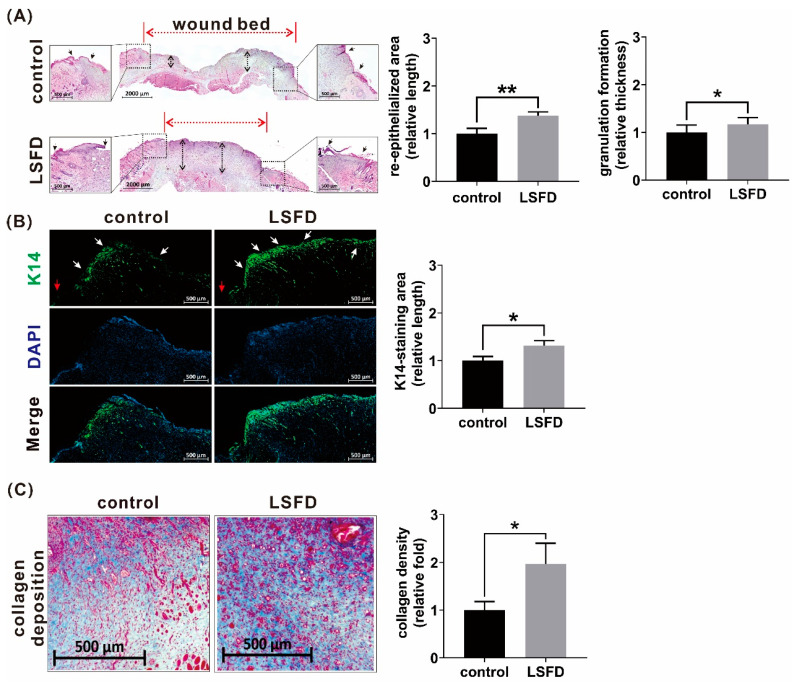
Histological characterization of rat burn wounds. (**A**) Hematoxylin and eosin staining of β-sheet LSFD-treated and control group tissue at day 10 post-wounding, showing the length of the re-epithelialized area and the thickness of the granulation tissue formation area. Black arrows: the distance from the original wound edges to re-epithelialized edges; black dashed arrows: granulation thickness; red dashed arrows: wound bed. (**B**) K14-immunofluorescence staining of epithelial tongues at day 10 post-wounding. White arrows: epithelial tongue area; red arrows: original wound edges; DAPI, 4′,6-diamidino-2-phenylindole (**C**) Masson’s Trichrome staining showing collagen deposition in rat skin at day 10 post-wounding. Quantitative data are presented as the mean ± standard error of the mean (*n* = 6; * *p* < 0.05, ** *p* < 0.01).

To determine whether β-sheet LSFDs accelerate re-epithelialization and granulation formation, day-10 wound tissue sections were analyzed using histological staining. The results of H&E staining showed a significant increase in the re-epithelialized distance in the LSFD group at day 10 vs. the control group. As for granulation tissue formation, a significantly thicker dermis was observed at day 10 in the LSFD group compared with the control group (Figure 3A). K14, a type of structural filament, is a biomarker of the epithelial tongue during re-epithelialization [40]. Thus, epithelial tongues were detected using a K14-immunofluorescence assay. The LSFD group showed a significantly longer distance of K14-staining area at day 10 vs. the control (Figure 3B). Collagen deposition is an essential foundational healing process during granulation tissue formation [41]. The collagen density on day 10 was significantly higher in the LSFD group than in the control group (Figure 3C). Therefore, β-sheet LSFD induced re-epithelialization and granulation formation during the proliferation phase of cutaneous wound healing.

### 3.4. β-Sheet SF Promoted In Vitro Migration of HDFs and HaCaT Keratinocytes

**Figure 4:**
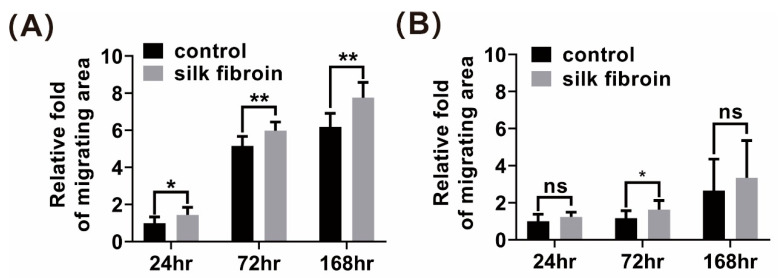
Influence of β-sheet silk fibroin on in vitro cell migration, expressed as the relative fold change in the migrating area of (**A**) human dermal fibroblasts and (**B**) HaCaT keratinocytes. Data are presented as the mean ± standard error of the mean (*n* = 4; * *p* < 0.05, ** *p* < 0.01, ns: not significant).

The effect of β-sheet LSFD on re-epithelialization and granulation formation during wound repair was confirmed. This indicates that keratinocytes and fibroblasts are attracted to the wound bed during tissue regeneration [42]. We investigated whether SF accelerated cell migration. To verify the cell recruitment abilities of SF, we used primary cultured HDFs and HaCaT keratinocytes. Fibroblasts in the SF-treated group showed a larger migrating area than the control group fibroblasts at 24, 72, and 168 h (Figure 4A), whereas SF-treated HaCaT keratinocytes only differed significantly from the control group at 72 h (Figure 4B). The tendency of SF to promote fibroblast migration was thus proven in vitro. We subsequently focused on granulation tissue formation in vivo and fibroblasts in vitro.

### 3.5. β-Sheet LSFD Promoted ECM Protein Expression in Rat Dermis

**Figure 5:**
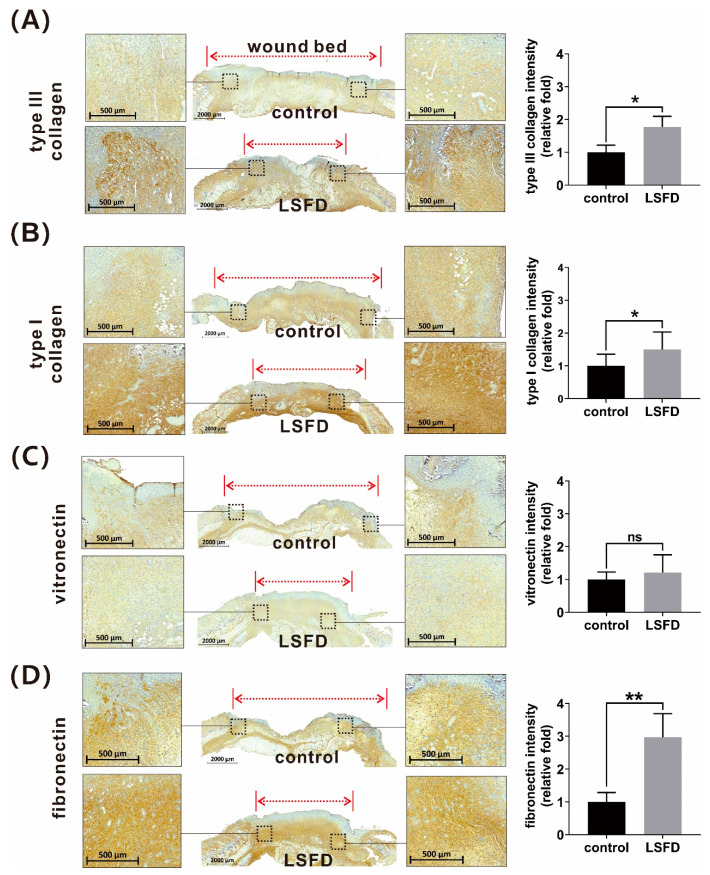
Immunohistochemistry staining and quantitative data of (**A**) type III collagen, (**B**) type I collagen, (**C**) vitronectin, (**D**) fibronectin rat tissue sections of the β-sheet lyophilized silk fibroin dressing (LSFD)-treated group and the control group. All quantitative representations show the relative fold difference in ECM protein expression between the β-sheet LSFD-treated group and the control group. Data are presented as the mean ± standard error of the mean (*n* = 6; * *p* < 0.05, ** *p* < 0.01, ns: not significant).

Collagen and other ECM proteins are responsible for granulation tissue formation in the dermis [43,44]. Our data showed that β-sheet LSFD promoted collagen deposition in the rat dermis (Section 3.3; Figure 3C). We further investigated whether β-sheet LSFD reconstructs dermal tissue by promoting several components of ECM proteins. IHC staining showed a significant increase in type III collagen, type I collagen, and FN expression in the LSFD group at day 10 post-wounding vs. the control group (Figure 5A,B,D, respectively); vitronectin expression was not significantly different from the control group (Figure 5C). It is evident that β-sheet LSFD promoted cutaneous regeneration partially by modulating the protein composition of the ECM.

### 3.6. Potential Mechanisms of β-Sheet LSFD Related to Cell Adhesion Molecules

**Figure 6:**
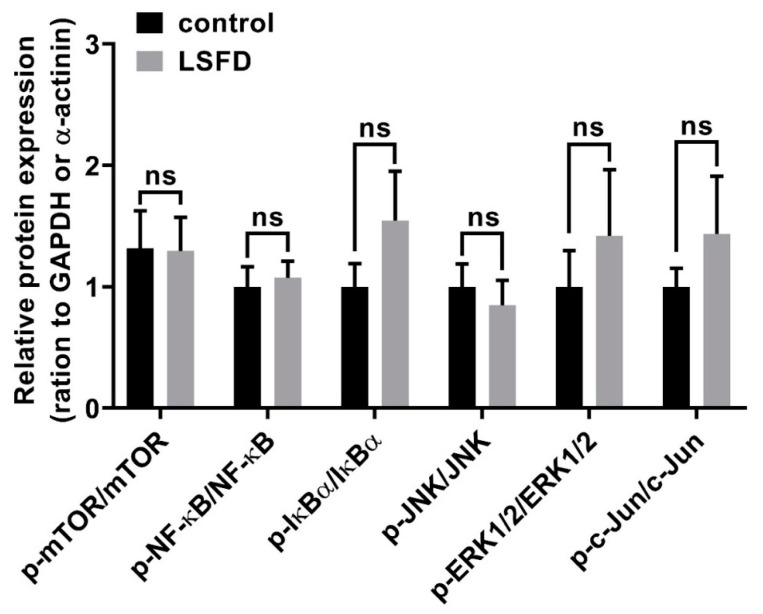
Western blot analysis data showing the relative protein expression in the wound bed at day 10 post-wounding in the control group and β-sheet LSFD-treated group. GAPDH, glyceraldehyde 3-phosphate dehydrogenase IκBα, nuclear factor of kappa light polypeptide gene enhancer in B-cells inhibitor, alpha; JNK, c-Jun N-terminal kinase; mTOR, mechanistic target of rapamycin; NF-κB, nuclear factor kappa B; p indicates the phosphorylated protein. Data are presented as the mean ± standard error of the mean (*n* = 7; ns: not significant).

Previous studies demonstrated that soluble SF treatment in fibroblasts could significantly increase the gene expression of cell adhesion molecules, mTOR, and NF-κB signaling pathways, which are involved in environmental information processing and promote cell migration [26]. Furthermore, human mammary gland and mink lung epithelial cell lines treated with soluble SF showed elevated expression of ERK1/2, JNK, and c-Jun, which are related to cell migration [45]. However, the mechanism by which β-sheet SF promotes fibroblast migration remains unknown and requires reliable evaluation in animal models. Because Western blotting of the homogenized wound bed tissue on day 10 showed no significant correlations between the phosphorylation of mTOR, NF-κB, IκBα, ERK1/2, JNK, and c-Jun between the LSFD and control group (Figure 6), therefore we focused on the connection between ECM proteins and integrins during β-sheet SF treatment to explore other potential mechanisms. Our tissue section staining showed the upregulation of ECM proteins in the LSFD group (Figure 5). Because integrins are ECM protein-binding receptors in cell adhesion molecules, they respond to cell motility and the actin cytoskeleton during wound repair [46]. Considering our observations, we hypothesize that SF may partially modulate integrin activity during dermal reconstruction.

### 3.7. β-Sheet LSFD Modulated ECM-Specific Integrin Expression in Rat Dermis

**Figure 7:**
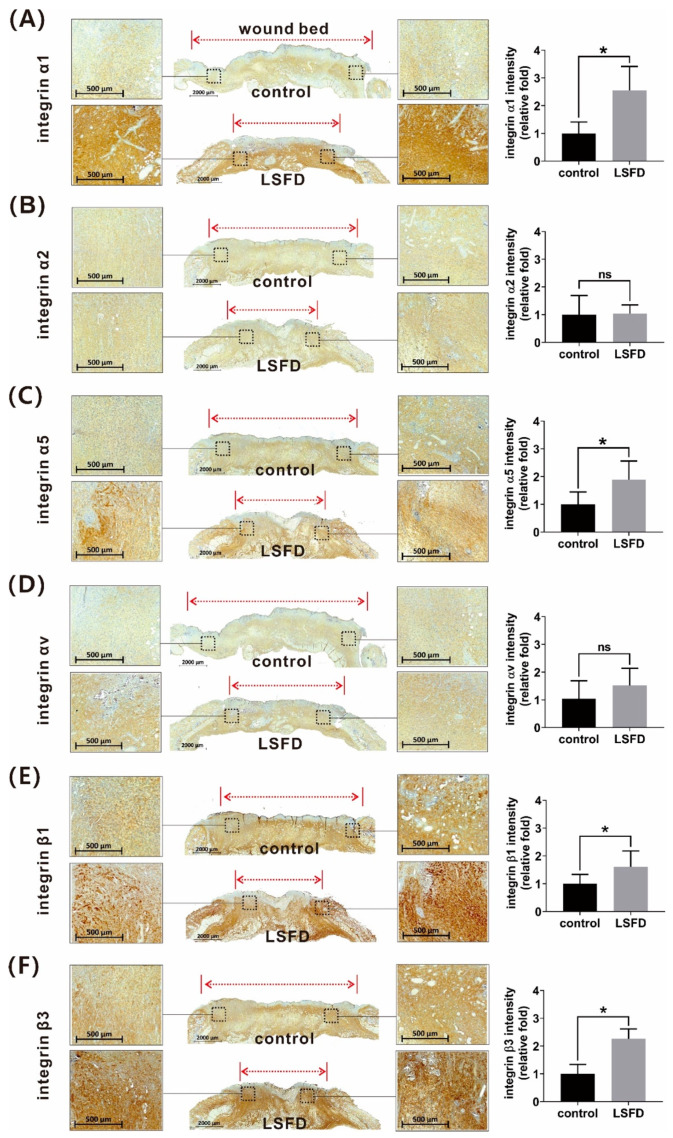
Immunohistochemistry staining and quantitative representations of the relative fold difference in the expression of different integrins in rat burn wounds treated with β-sheet lyophilized silk fibroin dressing (LSFD) and untreated burn wounds (control); (**A**) integrin α1; (**B**) integrin α2; (**C**) integrin α5; (**D**) integrin αv; (**E**) integrin β1; (**F**) integrin β3. Data are presented as the mean ± standard error of the mean (*n* = 6; * *p* < 0.05).

Considering our targeted ECM proteins, specific integrins for collagen (integrin α1, α2, and β1), vitronectin, and FN (integrin α5, αv, β1, and β3) were evaluated using IHC staining. Notably, the β-sheet LSFD-treated group showed an increase in integrin α1, α5, β1, and β3 expression at day 10 (Figure 7A,C,E,F). However, there was no significant difference in integrin α2 and αv expression between the LSFD and control groups (Figure 7B,D). Based on these observations, we inferred that SF may modulate cell adhesion molecules by topically facilitating integrin expression during dermal regeneration in burn wound healing.

### 3.8. β-Sheet LSFD Increased Fibroblast Differentiation and MMP-12 Activity

**Figure 8:**
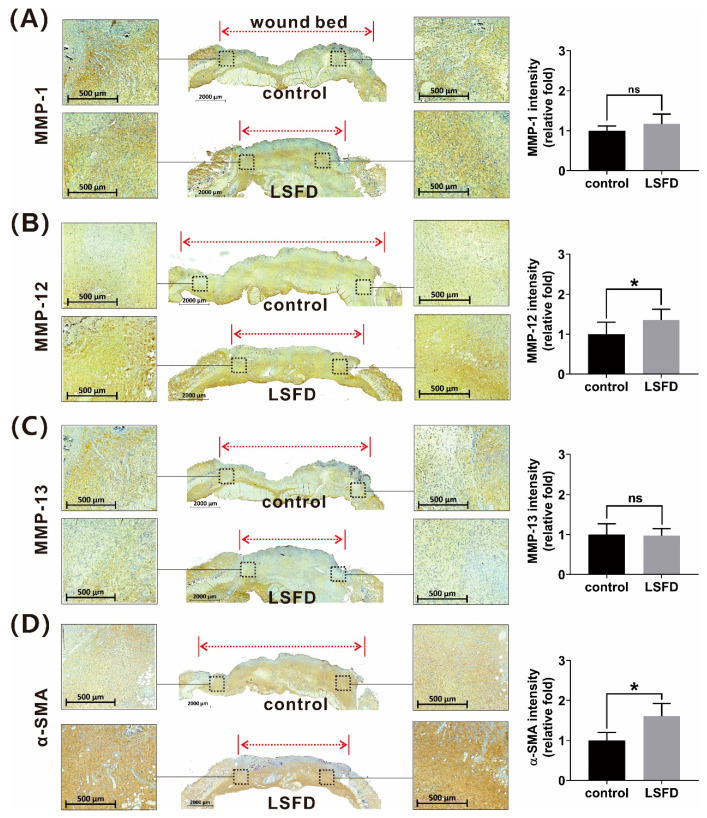
Immunohistochemistry staining of (**A**) matrix metalloproteinase-1 (MMP-1), (**B**) MMP-12, (**C**) MMP-13, and (**D**) α-smooth muscle actin (α-SMA) in day-10 tissue sections of untreated burn wounds (control) and burn wounds treated with β-sheet lyophilized silk fibroin dressing (LSFD). Quantitative representations show the relative fold difference in integrin and MMP expression between the LSFD-treated group and the control group. Data are presented as the mean ± standard error of the mean (*n* = 6; ns: not significant).

The downstream functions of integrin include the regulation of the cytoskeleton and MMPs, which, respectively, are involved in fibroblast differentiation and cell motility [47]. The fibroblast differentiation marker, α-SMA, has been shown to modulate wound contraction [48]. MMPs can specifically degrade ECM proteins and promote cell motility [49]. We speculated that β-sheet LSFD may modulate α-SMA and MMPs in the dermal layer network during burn wound healing. IHC staining and the *t*-test analysis indicated a significant difference in α-SMA and MMP-12 expression between the β-sheet LSFD-treated group and the control group. However, there were no significant differences on day 10 between the two groups in the expression of MMP-1 and MMP-13 in granulation tissue (Figure 8).

### 3.9. β-Sheet SF Stimulated In Vitro Expression of Integrin β1 and ECM Proteins in HDFs

**Figure 9:**
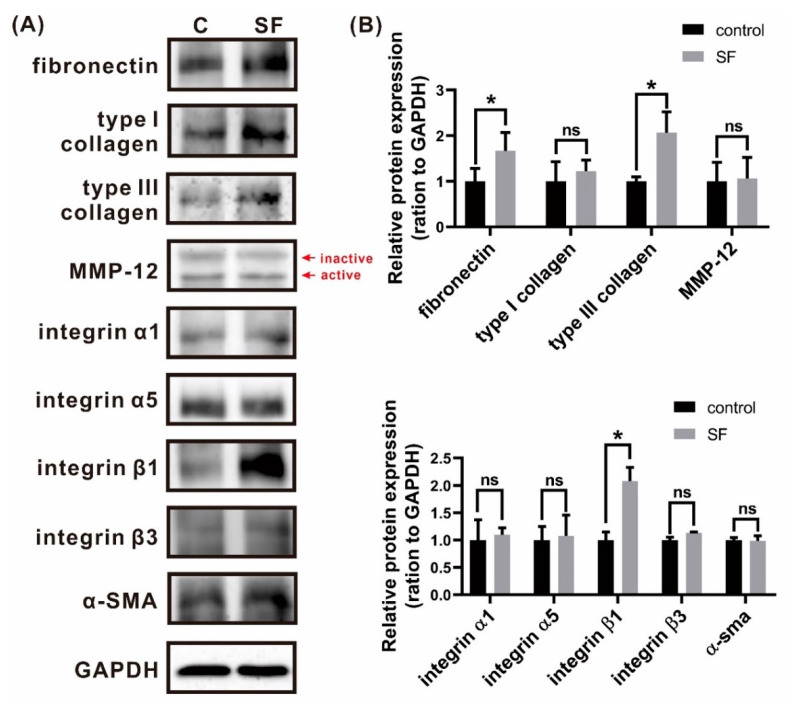
The protein expression in control and SF (silk fibroin) groups at 168 h were detected using Western blot analysis. (**A**) Fibronectin, type I collagen, type III collagen, MMP-12, integrin α1, α2, β1, β3, and α-SMA. (**B**) Quantitative representations showed the relative expression in the control and SF groups. Data are presented as the mean ± standard error. * *p* < 0.05, ns: not significant). α-SMA, alpha-smooth muscle actin; GAPDH, glyceraldehyde 3-phosphate dehydrogenase; MMP-12, matrix metalloproteinase-12.

To evaluate integrin and ECM protein expression in β-sheet SF-treated burn wounds, we utilized primary cultured HDFs to explore the mechanism of cell migration. HDFs treated with β-sheet SF exhibited increased expression of FN and type III collagen, but without the modulation of type I collagen and MMP-12. Additionally, β-sheet SF further promoted the expression of integrin β1, but not integrin α1, α5, β3, and α-SMA (Figure 9A). Our in vitro results indicate that β-sheet SF specifically promoted the expression of FN, type III collagen, and integrin β1 (Figure 9B).

## 4. Discussion

As a biomaterial with stable physical and chemical properties, insoluble SF in β-sheet form has been developed into multiple types of wound dressings. In the past, studies rarely reported the underlying mechanism of β-sheet SF during cutaneous wound healing [15]. Here, we showed that LSFD, which mainly consisted of β-sheet SF, promoted granulation tissue formation and re-epithelialization in an in vivo rat burn model and enhanced the motility of HDFs in vitro by increasing the expression of ECM proteins and integrin.

According to our animal experiments, LSFD enhanced cutaneous wound healing during the proliferation phase, including granulation tissue formation and re-epithelialization. These two critical steps are essential for skin regeneration. Our results are consistent with those of previous studies that used SF dressing to improve wound healing [20,50,51]; however, previous studies did not focus on the influence of β-sheet SF. This study used 75% ethanol treatment to achieve an α-helix to β-sheet SF transition. Based on the findings from HDFs and HaCaT cell lines, we speculate that LSFD promoted fibroblast migration and granulation tissue formation, which may assist in re-epithelialization through cell interactions.

Our results showed that β-sheet SF stimulated the expression of FN and type III collagen in HDFs in vitro and granulation tissue formation in vivo. FN and type III collagen, major ECM elements, are involved in cell migration and adhesion during tissue repair and correspond to specific integrins and MMPs [52,53,54]. Interestingly, type I collagen was significantly upregulated in vivo, but not in vitro. As type III collagen can be converted into type I collagen during cutaneous healing [54], LSFD may contribute to type I collagen synthesis by initially regulating type III collagen expression.

It is well known that MMPs secreted by dermal fibroblasts facilitate the migration of surrounding fibroblasts and keratinocytes [55,56], and that MMP-12 is responsible for the degradation of type I and III collagen [57,58]. In this study, LSFD increased MMP-12 expression in granulation tissue; however, the in vitro results showed that β-sheet SF did not influence MMP-12 expression. As MMP-12 can be secreted by both fibroblasts and macrophages [59,60], LSFD may modulate the macrophage response to MMP-12 secretion in the repaired wound bed. However, the interaction between β-sheet SF and macrophages requires verification.

Park et al. examined the gene levels of soluble SF-treated fibroblasts and observed that soluble SF moderated biomarkers of environmental information processing, including cell adhesion molecules [26]. Soluble SF further possesses an underlying mechanism involving phosphorylation kinases, including mTOR, NF-κB, IκBα, ERK1/2, JNK, and c-Jun [26,45,61]. However, significant phosphorylation levels of these kinases were not observed in the wound bed granulation tissue in this study. The divergence in SF treatment compared with previous research may be due to the SF forms, which differed from the β-sheet SF used in our experiments. These results indicate that soluble and insoluble SF may possess different migration mechanisms.

As for β-sheet forms of SF, Tan et al. demonstrated that insoluble SF induced integrin expression in endothelial cells, which participated in cell motility [29,62]. Therefore, we are interested in integrins, which belong to the receptors of ECM proteins and regulate cell migration and fibroblast differentiation [63,64]. Integrin α5β1 and αvβ3 can bind with fibronectin [65] and play crucial roles in wound healing [66,67,68], while cell adhesion to collagen is mediated by integrin α1β1 [69]. The β subunits form heterodimers with α integrins that can promote cell migration, which has been reported to participate in the development of cancer [70,71], the immune system [72], and in tissue repair [73,74]. β1 knockout in fibroblasts reduced cell functions such as cell adhesion, spread, differentiation, expression of collagen and connective tissue growth factor, cutaneous wound closure, and ECM deposition [74]. Interestingly, α1, α5, β1, and β3 subunits were significantly upregulated in our in vivo study, while only the β1 subunit was upregulated in HDFs in vitro. Additionally, LSFD activated the expression of α-SMA, a biomarker of fibroblast differentiation, in vivo, but not in vitro. The inconsistency between the in vivo and in vitro results may be due to the complexity of the microenvironment during wound healing, which could influence α1, α5, and β3 subunits and regulate fibroblast differentiation. The detailed working mechanisms of soluble and insoluble SF may be another issue for further investigation.

Our in vivo and in vitro results showed that LSFD is beneficial for tissue regeneration. Besides, LSFD is an ideal matrix for bringing small molecules or substances with weak mechanical properties to the living body, such as a gene delivery system for bone regeneration [75]. Moreover, the β-sheet form SF scaffold also could also provide a cancer cell migration system for studying cancer metastasis [76]. There were many kinds of SF wound dressing developed in the labs; however, the clinical trials of LSFD are still lacking [77]. Until 2017, Zhang et al. verified that silk fibroin film, compared with commercial wound dressing, elevated wound healing in a randomized controlled clinical trial [78]. Therefore, the healing effect of LSFD under ethanol treatment should be more validated in clinical trials.

Because SF possesses good mechanical strength, is non-cytotoxic, and does not cause inflammation and skin sensitivity, it is very suitable as a biomaterial for wound dressing [15]. The porosity of LSFDs facilitates moisture absorption [79], allowing the burn wound exudate to be readily absorbed. Our animal experiments have also shown that LSFD can keep the wound bed moist while not sticking to the wound, creating a favorable microenvironment for wound healing. Studies have indicated that the degradation rate of silk with high β-sheet content is low [80], thus it maintains a stable structure on the wound bed. Hydrogel dressings or other easily degraded biological materials lack this property. LSFD produced by lyophilization and ethanol treatment can be easily prepared on a large scale. However, after ethanol-treated SF is dried at room temperature, the texture becomes brittle [81]. This problem needs to be addressed in future studies. Fortunately, the mechanical properties of the β-sheet SF material can be adjusted by applying post-processing technologies [82]. By combining with other materials or adjusting the processing method, the softness and flexibility of the dressing can be improved to meet clinical requirements [83].

There are some limitations in this study. Because many commercial antibodies can be used in rats for biomolecular mechanism research, we chose rats for this animal experiment, which is also the species chosen by many scholars for animal studies of wound healing. However, the anatomical structure of rat skin is partially different from human skin, and the wound area that can be created in a rat is limited. Therefore, the severity of burns in rats is challenging to be categorized with the general scald grading of humans directly, and the current conclusion cannot be directly inferred to human skin and to more extensive wounds. Compared with other species, porcine skin is structurally closest to humans [84]. Hence, LSFD should be investigated in porcine models for future preclinical animal studies.

**Figure 10:**
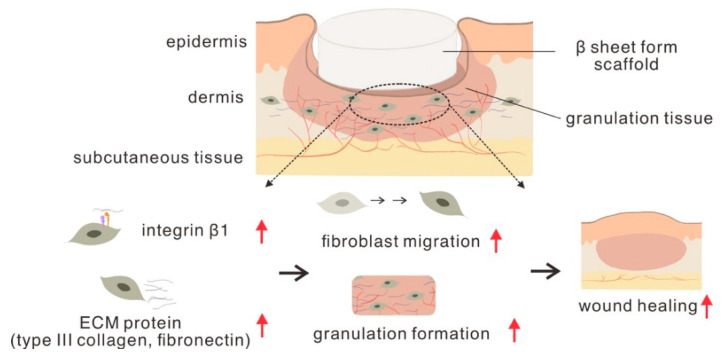
Schematic illustration of the summary in this study.

In conclusion, this study demonstrated that β-sheet SF increased dermal fibroblast migration and upregulated the protein expression of ECM (fibronectin and type III collagen) and integrin β1 subunit. This may promote granulation tissue formation, increase wound re-epithelialization, and finally improve burn wound healing (Figure 10). Our results suggest that LSFD is a potential therapeutic strategy for promoting wound repair and regeneration. LSFD could potentially be combined with anti-inflammatory materials, growth factors, or antibiotic reagents to develop various novel dressings with ideal qualities for tissue regeneration.
